# Liver Injury in Patients with Coronavirus Disease 2019 (COVID-19)—A Narrative Review

**DOI:** 10.3390/jcm10215048

**Published:** 2021-10-28

**Authors:** Liliana Łykowska-Szuber, Karolina Wołodźko, Anna Maria Rychter, Aleksandra Szymczak-Tomczak, Iwona Krela-Kaźmierczak, Agnieszka Dobrowolska

**Affiliations:** Department of Gastroenterology, Dietetics and Internal Diseases, Poznan University of Medical Sciences, 60-355 Poznań, Poland; karolina.wolodzko@gmail.com (K.W.); aleksandra.szymczak@o2.pl (A.S.-T.); krela@op.pl (I.K.-K.); agdob@ump.edu.pl (A.D.)

**Keywords:** COVID-19, liver disease, clinical manifestations

## Abstract

While respiratory symptoms are prevalent in SARS-CoV-2 infected patients, growing evidence indicates that COVID-19 affects a wide variety of organs. Coronaviruses affect not only the respiratory system, but also the circulatory, nervous and digestive systems. The most common comorbidities in COVID-19 patients are hypertension, followed by diabetes, cardiovascular, and respiratory disease. Most conditions predisposing to SARS-CoV-2 infection are closely related to the metabolic syndrome. Obesity and chronic diseases, including liver disease, are associated with the induction of pro-inflammatory conditions and a reduction in immune response disorders, leading to the suspicion that these conditions may increase the susceptibility to SARS-CoV2 infection and the risk of complications. The definition of liver damage caused by COVID-19 has not yet been established. COVID-19 may contribute to both primary and secondary liver injury in people with pre-existing chronic disease and impaired liver reserves, leading to exacerbation of underlying disease, liver decompensation, or acute chronic liver failure. Therefore, many researchers have interpreted it as clinical or laboratory abnormalities in the course of the disease and treatment in patients with or without pre-existing liver disease. The research results available so far indicate that patients with liver disease require special attention in the event of COVID-19 infection.

## 1. Introduction

It has been over a year since China reported first cases of a mysterious new strain of pneumonia to the World Health Organization and 18 months since it was declared pandemic. We are now vaccinating patients with the fastest vaccine ever developed. The International Committee on Taxonomy of Viruses named the new virus severe acute respiratory distress syndrome coronavirus-2 (SARS-CoV-2) and the condition caused by it, coronavirus infectious disease 2019 (COVID-19) [1]. By the time of this article, WHO has reported over 220 million confirmed cases of COVID-19 of which 55% have fully recovered and 2% have died [2,3].

Although respiratory symptoms with widely varying severity dominate in patients infected with SARS-CoV-2, a growing amount of data highlight the impact of COVID-19 on multiple organs, as it was in the case of SARS-CoV and MERS-CoV. Liver impairment, mainly in the form of biochemical abnormalities, has also been frequently reported as a common manifestation. It is important to determine the clinical and prognostic significance of these disorders and the implications of this new disease for patients with pre-existing liver diseases, such as viral hepatitis, alcoholic liver disease, non-alcoholic fatty liver disease (NAFLD), autoimmune hepatitis and cirrhosis.

## 2. Clinical Manifestations of COVID-19

SARS-CoV-2 is an enveloped positive-sense single-stranded RNA virus that belongs to the Coronaviridae family and Betacoronavirus genus [4]. Numerous coronaviruses have been found in animals, but only seven are pathogenic to humans, including three that have caused major epidemics of severe pneumonia in the previous two decades. SARS-CoV induced an outbreak of severe acute respiratory syndrome (SARS) in China in 2002. MERS-CoV was identified as the cause of Middle East respiratory syndrome (MERS) in 2012 [4].

Since SARS-CoV-2 belongs to the same Coronaviridae family and its genome sequence is approximately 80% homologous to SARS-CoV, and 50% to MERS-CoV, they share similarities in structure and pathogenicity [4].

All three coronaviruses affect not only the respiratory tract, but also the cardiovascular, nervous and digestive systems.

Most patients present mild symptoms, such as fever, fatigue, dry cough, and myalgia, accompanied by less common symptoms: diarrhea, vomiting and loss of the sense of taste or smell. Symptoms of severe disease—dyspnea and signs of hypoxemia, usually occurring a week after onset of illness—indicate severe pneumonia that can lead to acute respiratory distress syndrome (ARDS), multiple organ dysfunction syndromes (MODS) and death [5].

After an outbreak of severe acute respiratory syndrome (SARS) in 2002, research on the pathogenesis of SARS-Cov revealed that the virus was internalized into the host cells through the functional angiotensin-converting enzyme 2 receptor (ACE2) [6,7]. The same receptor is functional for the novel coronavirus, as both betacoronaviruses belong to genetic lineage B [7]. The virion’s envelope spike consisting of glycoprotein (S protein), specifically its receptor-binding domain (RBD), is a ligand that binds with the host cell surface ACE2 receptor facilitating membrane fusion, virus entry and replication [7,8]. However, SARS-Cov-2 binds to the ACE2 with 10-fold to 20-fold higher affinity, compared to SARS-CoV, which may explain its fast transmission rate among humans [9].

The target receptor is highly expressed in lung type II pneumocytes, but prevalence of the extra-pulmonary symptoms suggests that other organs may be also affected by SARS-CoV-2. Transcriptomics and immunohistochemistry studies have proved the presentation of the highest proportion (>1%) of the ACE2 receptor in the lower respiratory tract, lungs, heart, ileum, esophagus, kidneys, and bladder. Organs with lower ACE2 expression levels are, among others, the liver, stomach, pancreas, brain, vessels endothelium, testis, uterus, ovary, breast, oral and nasal mucosa [10].

## 3. COVID-19 and Comorbidities

COVID-19 affects all age groups with a median age of 47 years, and it is more prevalent in men and those with comorbidities [11].

Moreover, older age and the presence of additional disease were more common among patients with severe course of infection.

A meta-analysis involving 46,248 patients showed that the most common comorbidities in COVID-19 patients are hypertension (14–22%), followed by diabetes mellitus (6–11%), cardiovascular diseases (4–7%) and respiratory disease (1–3%). The same conditions are associated with higher mortality [12]—49% in comparison to overall case fatality rate at 2–5% [11,12].

Three studies from New York associated overweight and obesity with higher prevalence of COVID-19 and greater risk of hospitalization in intensive care units [13,14,15].

Most of the conditions predisposing to SARS-CoV-2 infection are closely related to metabolic syndrome. The liver, as an organ crucial for lipid and glucose metabolism, is a key determinant of metabolic abnormalities. Therefore, it is not surprising to observe that some research linked severe course of COVID-19 to non-alcoholic fatty liver disease (lately referred as metabolic dysfunction-associated fatty liver disease—MAFLD [12]), which is likely seen as a cause or consequence of metabolic syndrome [13]. It remains unclear whether the risk is specific to NAFLD or results from coexisting metabolic conditions.

Obesity and chronic disorders, including liver diseases, are associated with the induction of proinflammatory states and the attenuation of disturbances in the immune response, leading to suspicion that these conditions may increase the susceptibility to SARS-CoV2 infection as well as the risk of complications.

## 4. COVID-19 and Liver Disease

A review of 12,882 confirmed COVID-19 patients hospitalized until the end of June 2020 showed that overall prevalence of hepatic comorbidities in this group was 2–11% and it was not associated with poorer outcomes of infection [14,16]. However, according to an American study focusing on patients with preexisting liver diseases (9% of 2780 individuals), they are at higher risk of death (12% vs. 4%) and increased hospitalization days [15]. However, in this group, the percentage of mortality was the highest. It is also worth noting that in this cohort, patients with underlying hepatic conditions were older with a larger proportion of other comorbidities, such as hypertension (68%) or diabetes (48%)—even in comparison to data from the meta-analysis mentioned above [15].

The hepatic distribution of the SARS-CoV-2 target receptor discussed above is heterogeneous. Many reviews have confirmed its presence on cholangiocytes and absence on Kupffer cells, or sinusoidal endothelial cells [16,17].

Hamming and colleagues, who used immunohistochemistry for evaluation, reported that hepatocytes are negative for the ACE2 receptor [18]. However, other studies performed with the use of scRNA-seq identified low frequency of ACE2 expression on the same cells [19,20]. Moreover, in the same research, Chai et al. showed that the level of ACE2 expression cholangiocytes was found to be similar to that of type II pneumocytes [19]. These findings suggest that hepatocytes might not be targeted directly by the virus, contrary to the biliary epithelium. The finding does not explain why the majority of patients with COVID-19-related liver injury tend to present with abnormal AST and/or ALT, but not a cholestasis picture.

The definition of COVID-19-induced hepatic damage has not been established yet. That is why many researchers interpret it as any liver-related clinical or laboratory abnormality occurring during the course of the disease and treatment in patients with or without pre-existing liver condition.

Similar to SARS and MERS, patients have shown various degrees of liver impairment, most commonly presented as mild-to-moderate aminotransferase elevation, in some cases accompanied by a slight increase in serum bilirubin and decrease in albumin levels [21]. Disfunction of the liver among COVID-19 patients ranged from 14.8% up to 53% and is significantly higher in patients with a more serious infection, reaching 58–78% among death. Coincidence of hypoalbuminemia has been reported to be an independent predictive factor for poor prognosis [22]. The predominant type of liver injury is the hepatocellular pattern. It is worth mentioning that damage of skeletal or cardiac muscle, also occurring in COVID-19, could also result in the elevation of serum transaminase along with LDH levels.

According to the study by Kaneko S. et al. the predictors of liver impairment presented by transaminases elevation are C-reactive protein (CRP) at baseline, oxygenation, intubation, and gastrointestinal symptoms, such as appetite loss, diarrhea and nausea [23]. This confirmed conclusions from a couple months before when Jin X et al. had found that liver impairment occurred more frequently in patients presenting gastrointestinal signs. The same research showed that the coexistence of underlying liver disease also is associated with digestive tract symptoms [24].

In retrospective studies, it was also observed that low testosterone concentration in younger men (<65 years of age) may be an independent factor predisposing to acute liver failure in the course of COVID-19. In people over 65 years of age, the gender difference did not affect the occurrence of this complication [25,26]. It may result from the fact that testosterone deficiency is associated with obesity, metabolic syndrome, type 2 diabetes and their clinical consequences, such as fatty liver and atherosclerosis. In younger women, female sex hormones are protective in this regard [27].

**Figure 1:**
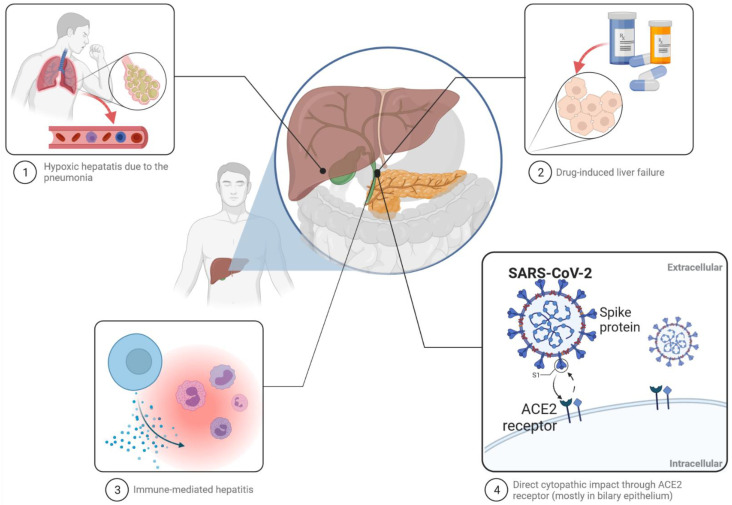
Probable pathogenesis of liver injury caused by SARS-CoV-2.

The pathogenesis of liver injury caused by SARS-CoV-2 is still unclear. Several possible mechanisms are taken under consideration: direct cytopathic effect of the virus through the ACE2 receptor, immune-mediated hepatitis as a result of uncontrolled inflammatory response following COVID-19 infection, leading to cytokine storm syndrome, anoxia as a cause of hypoxic hepatitis due to pneumonia and respiratory failure and drug-induced liver injury secondary to medications used for the treatment, among others, as well as antipyretic drugs or antiviral agents (Figure 1) [19,28,29,30].

### The Mechanisms of Liver Injury in COVID-19

It can be suspected that a combination of these mechanisms results in the acute liver injury observed in COVID-19 patients, especially in those with a severe course of the disease, experiencing severe pneumonia, SIRS, sepsis or MODS.

Moreover, COVID-19 may contribute to additional hepatic impairment in people with pre-existing chronic liver conditions and compromised hepatic reserves, leading to exacerbation of the underlying disease, hepatic decompensation or acute-on-chronic liver failure.

Post-mortem examination of 44 cases revealed signs of a pre-existing liver disorder in 18 cases (*n* = 14 steatosis, and *n* = 4 cirrhosis) [31]. Liver histology results have varied depending on the study. The main findings were congestive hepatopathy and moderate microvascular steatosis. In some cases, patchy hepatic necrosis, mild lobular lymphocytic infiltration and nuclear glycogen deposition were reported [31,32]. None of the findings are specific for direct viral injury, even though the presence of viral RNA was detected through RT-PCR in the liver parenchyma, as it was in the case of the SARS-CoV genome [32,33].

Currently, there are insufficient data regarding the impact of SARS-CoV-2 infection on the course of pre-existing chronic liver conditions. As mentioned above, the overall prevalence of hepatic comorbidities among COVID-19 patients was reported to reach 2–11% with a predominance of NAFLD (MAFLD), whose presence increased the risk of disease progression, abnormal liver function and longer viral shedding time, compared to patients without NAFLD [15]. In another study, MAFLD was associated with more than 2-fold higher prevalence of severe infection, but only in patients younger than 60 years. The mechanisms of the age-dependent relationship are unclear (See Table 1) [16].

Another risk factor for COVID-19 exacerbation is the presence of fibrosis in MAFLD/NAFLD, established with the use of the non-invasive evaluation of FIB-4 or nonalcoholic fatty liver disease fibrosis (NFS) scores, independently of metabolic comorbidities [34].

A case-control study from the U.S.A. confirmed that patients with chronic liver disease had significantly higher odds of developing COVID-19, compared to those without hepatic comorbidity, even after adjusting for COVID-19 risk factors (however, authors showed that age and gender had no additional effect on the risk of acquiring COVID-19 among patients with chronic liver disease, in contrast to the race). The strongest correlation concerned non-alcoholic liver disease and non-alcoholic cirrhosis, followed by patients with chronic hepatitis C, alcoholic liver damage, alcoholic liver cirrhosis, and chronic hepatitis B. Furthermore, patients with both SARS-CoV-2 infection and pre-existing liver disease had higher rates of hospitalization and death than others [35].

Cirrhotic patients were reported to be more susceptible to the novel coronavirus infection, which has led, in almost half of cases, to hepatic decompensation of cirrhosis and eventually to the development of acute-on-chronic liver failure, due to potential impairment of the innate immune system in encounters with a cytokine storm [36,37]. Additionally, the mortality rate is higher in COVID-19 patients with alcohol-related liver disease and cirrhosis, increasing in line with the Child–Pugh class (A [19%], B [35%], C [51%] reaching up to 100% in patients presenting as acute-on-chronic liver failure [36,37]. However, the main cause of death is respiratory failure [37]. Therefore, the suggestion that cirrhosis patients presenting with signs of decompensation of the liver disease should be evaluated for SARS-CoV-2 infection is advisable (Table 1) [36].

After a year of pandemic, data on correlation between COVID-19 and less common chronic liver diseases are limited. A few studies concerning AIH patients have shown that incidence of SARS-CoV -2 infection in this group was similar to that of the general population [38]. Moreover, adverse outcomes, such as hospitalization, ICU admission, mechanical ventilation and death, are comparable between AIH patients and those with other chronic liver diseases, including PSC and PBC patients, or without any hepatic comorbidity [39]. Baseline stadium of cirrhosis and age are independent determinants of death, while immunosuppressive therapy is not associated with an increased risk of severe course of infection or mortality [39,40].

## 5. SARS-CoV-2 Infection and Liver Transplantation

Another population affected by the pandemic are liver transplant patients. Management of waiting list candidates and post-transplant patients as well as the possibility of performing surgical procedures have been impeded for over one and a half years, due to limitations on elective and non-urgent surgery, and the reduced availability of hospital and intensive care beds, medical staff, blood products and personal protective equipment, but also uncertainty about the safety of transplantation during the SARS-CoV-2 pandemic.

Analysis of 128 surveys completed by transplant centers from all over the world revealed a decrease in the number of performed liver transplants and wait-listed candidates with higher mortality among this group in comparison to 2019 [41].

The main symptoms of SARS-CoV-2 infection among this group are same as in the general population—fever, cough and shortness of breath. The incidence of gastrointestinal symptoms, such as diarrhea, was reported to be more common in post-transplant patients than in the control group [42,43].

Up to this point, it is not fully confirmed whether organ transplant recipients are at higher risk for severe course of COVID-19 or mortality. Based on several studies, it appears that liver transplantation is not an independent predictive factor for poor prognosis, but combined with immunosuppression, underlying diseases and elderly age increases that risk [44].

The presence of non-liver cancer, older age and higher baseline serum creatinine are factors associated with death [43].

As mentioned before, data collected during three coronavirus epidemics suggest that immunosuppression is not among the risk factors of severe course of the disease or higher mortality [45].

On the one hand, immunosuppressants used to reduce the risk of transplant rejection may increase susceptibility to COVID-19, but on the other, they potentially impair the uncontrolled inflammatory response following SARS-CoV-2 infection. Furthermore, long-term use of immunosuppressants may prolong the period of viral shedding and may, therefore, prolong the duration of the communicable period [46].

Another issue raises the question of donor-derived coronavirus infection. Although RNA of SARS-CoV, MERS-CoV and SARS-CoV-2 were detected in blood samples from a certain percentage of affected patients and residual SARS-CoV-2 viral antigens were found in the hepatic tissues of convalescents, no documented cases of coronavirus transmission through transplantation have been reported [47,48].

COVID-19 patients were not considered potential living or deceased donors, as long as the risk of virus transmission was not eliminated [49].

Most societies recommend the restriction of transplantation to patients with acute liver failure (ALF/ACLF), high model for end-stage liver disease (MELD) score or hepatocellular carcinoma (HCC) at the upper limits of the Milan criteria. Moreover, organ donors and recipients should be tested for SARS-CoV-2 [50].

## 6. Consensus World Gastroenterology Organization for the Care of Patients with COVID-19 and Liver Disease

As the pandemic spreads, our understanding of how the virus affects our patients grows. Knowing already the negative impact of SARS-CoV-2 virus on the liver and the risk of liver damage, many scientific societies have presented their views on the care of patients with liver diseases. Below, we present the position of the World Gastroenterology Organization (WGO). It is important to clarify when and which group of patients require liver function tests. The WGO states that in the absence of underlying liver disease, outpatients with COVID-19 do not require routine biochemical analysis. In contrast, all hospitalized patients should undergo laboratory evaluation, including ALT, AST, GGT, ALP, and bilirubin. It is unclear whether patients with HCV or HBV infection are at higher risk of liver damage from COVID-19. In this group of patients, antiviral therapy should not be interrupted, under no circumstances. Undoubtedly, patients with liver cirrhosis have a worse prognosis, including the risk of acute liver decompensation. Patients with autoimmune hepatitis, due to the immunosuppressive treatment, constitute a group of particularly high risk. During the SARS-CoV-2 infection, it is not recommended to reduce immunosuppressive therapy, as it may lead to exacerbation of the underlying disease and the need for glucocorticoids. However, in the course of COVID-19, lymphopenia is also observed in patients. In such a situation, a reduction in azathioprine dosage may be necessary. Patients with a poor short-term prognosis, e.g., patients with a high MELD score, acute liver failure, or HCC should be eligible for liver transplantation. Both donors and recipients should have rapid PCR tests for SARS-CoV-2 virus. Given the possible complications of COVID-19 for patients with liver disease, stable patients should be encouraged to telemedicine whenever possible. This will minimize the potential risk of infection [3].

## 7. COVID-19 Vaccinations in Patients with Liver Disease

The development of vaccines against SARS-CoV-2 has raised questions about their effectiveness and safety in the general population as well as in the population of patients with coexisting diseases. People with comorbidities require special attention in times of COVID-19 pandemic; therefore, vaccination prophylaxis is particularly important for them. Patients with underlying liver diseases may present a lower immune response to vaccination as was indicated in the previous studies on the response to vaccination against hepatitis A, hepatitis B and seasonal flu. Immunosuppressive drugs commonly used by autoimmune hepatitis patients contribute to a reduced response to vaccination [51,52,53].

The pace of the development of vaccines against SARS-CoV-2 has been undoubtedly unprecedented. According to the WHO COVID-19 vaccine tracer, since the beginning of pandemic up to now, 126 different vaccines have been included into clinical development. Seven of them have been approved by WHO for use. The most commonly administered are BNT162b2 (Pfizer-Bio NTech) and mRNA-1273 (Moderna), which are based on mRNA encoding SARS-CoV-2 spiny glycoprotein variants. The two remaining are adenoviral vector–based vaccines: ChAdOx1 nCov-19 (AZD1222, also known as the Oxford-AstraZeneca) is a replication-free chimpanzee adenoviral vector containing a full-length, codon-optimized gene encoding the SARS-CoV-2 spike protein; and Ad26.COV2.S (Johnson & Johnson/Janssen) is a recombinant human adenovirus type 26 vector encoding SARS-CoV-2 spike protein. None of the three vaccines contain any living virus and consequently cannot replicate, even in immunocompromised individuals [54]. Introducing a new vaccine is always accompanied by concerns about the occurrence of complications. The COVID-19 vaccines can cause mild side effects after the first or second dose; the main ones include temperature rise, general fatigue, and pain at the injection site, but in a small percentage of cases, severe adverse events, such as an anaphylactic shock, may occur [55]. Another issue regarding new vaccines is related to the potential ability to induce autoimmune conditions. Both patients and clinicians are concerned about the potential risk for relapse or worsening of autoimmune diseases mainly because of insufficient data. So far, such a correlation has not been unequivocally proven for any of the vaccines used [56]. It should be noted that clinical trials of vaccines also included patients with stable chronic liver disease. The data on COVID-19 vaccines in patients with chronic liver disease are limited, but until now, there was no higher frequency or severity of adverse events noted in these subgroups [57]. While we do not have data on the long-term safety of SARS-CoV-2 vaccinations, it is important to consider the risks of gains and losses when deciding whether to vaccinate. This is particularly important in the groups of patients where the risk of complications and mortality due to COVID-19 is much higher than in the general population. There is currently no approved commercial test to measure neutralizing antibody responses to SARS CoV-2. Therefore, we are not able to unequivocally assess the responses to the used vaccination. Research in this area is ongoing. Meanwhile, we should note that the benefits of authorized vaccines outweigh the potential risks of adverse events [58].

## 8. Consensus American Association for the Study of Liver Diseases and European Association the Study of the Liver on COVID-19 Vaccination

The American Association for the Study of Liver Diseases and European Association the Study of the Liver suggest that COVID-19 vaccines should be administered to all adult patients with chronic liver disease and liver transplantation. In addition, patients with chronic liver disease who receive antiviral drugs for hepatitis B or hepatitis C should not withhold their medications while receiving COVID-19 vaccines. For patients with hepatocellular carcinoma undergoing locoregional or systemic therapy, vaccination should also be considered without interruption in their treatment. For liver transplant candidates, COVID-19 vaccination should be continued, even if liver transplant occurs before the second dose is given. The second dose should be administered at the earliest appropriate time after transplantation (e.g., 6 weeks post-transplant). It is recommended that people with a known history of previous COVID-19 infection wait a minimum of 90 days before receiving a COVID-19 vaccine, due to concerns about an overly exaggerated immune response. However, there may be special circumstances under which prior vaccination may occur.

While co-administration of vaccines is usually safe, we do not have much data on whether co-administration reduces the immune response. Vaccinations against COVID-19 have become a challenge for contemporary global health protection. Given that flu season is approaching, researchers asked themselves whether it would be possible to safely administer both vaccines at the same time. This would undoubtedly reduce the costs of vaccinations and increase the number of vaccinations received. It is important to establish whether co-administration reduces the immune response to either vaccine, taking into account the importance of complete protection against both infections. During the COVID-19 pandemic, we realized the importance of cellular immunity and immune memory in evaluating vaccine responses, and antibodies are only one aspect of the immune response. Based on the currently available studies, it can be concluded that the concomitant administration of the influenza vaccine with either a booster dose or a second dose of COVID-19 vaccine is safe. More research is ongoing [59,60,61].

## 9. Conclusions

In summary, based on the observations so far of patients with a history of COVID-19, coexisting liver injuries are usually mild and do not require special treatment. In cases of severe hepatic injury reported in the literature, underlying liver disease or ischemic hepatitis is most likely to be present. It cannot be ruled out that the drugs used may influence the degree of liver damage. Their number in more severe course of infection is much greater [62].

Despite the fact that the amount of data on COVID-19 and its consequences is increasing every day, understanding of the long-term health effects of the disease and assessing its impact on patients with comorbidities require further research.

Studies on patients with underlying chronic liver diseases, referred to in this article, included small groups of individuals, given the total number of COVID-19 confirmed cases. In addition, it is difficult to separate liver disease patients from those with coexisting hypertension, cardiovascular disease, diabetes mellitus or obesity, which are known to be associated with COVID-19 increased susceptibility and risk of severe course of the disease. Therefore, larger studies with detailed evaluation and long-term follow-up will provide more information in this area.

However, the results of studies available so far indicate that patients with liver diseases require special attention in the case of COVID-19 infection [26]. At the same time, it should be remembered that patients at risk with chronic liver disease should be vaccinated against COVID-19 first.

## Figures and Tables

**Table 1:** Liver statistics and outcomes.

Data/Location	Statistics/Outcomes											
Sharma et al. meta-analysis 2020 [16]	Liver disease statistics											
	Acute LD			Comorbid chronic LD			Elevated AST			Elevated ALT		
	26.5%			2.6%			41.1%			29.1%		
	Poor outcomes, odds ratio											
	1.68			0.96 (insignificant)			1.85			2.98		
Singh et al. 2020 Multicenter Research Network Study, U.S.A. [17]	Mortality, relative risk											
	Preexisting liver disease						Cirrhosis					
	2.8						4.6					
Ji et al. 2020 China [15]	Liver disease statistics											
	Admission						Hospitalization					
	50%						75.2%					
	COVID-19 progression, odds ratio											
	Higher BMI						NAFLD					
	1.3						6.4					
Shalimar et al. 2020 India [36]	Liver disease statistics (total 3.7%, *n* = 28)											
	Liver cirrhosis, *n* = 26				NAFLD, *n* = 1				EHPVO, *n* = 1			
	Mortality, %											
	Cirrhosis						Without cirrhosis					
	42.3%						23.1%					
Marjot et al. 2021 International registry study, U.K. [37]	Mortality, %											
	Cirrhosis						Without cirrhosis					
	32.00%						8.00%					
	Mortality, odds ratio											
	Child–Pugh A			Child–Pugh B			Child–Pugh C			Cirrhosis and alcohol-related diseases		
	1.9			4.14			9.32			1.79		

LD—liver disease; AST—aspartate aminotransferase; ALT—alanine aminotransferase; BMI—body-mass index; NAFLD—non-alcoholic fatty liver disease.

## Data Availability

Data are available and publicly accessible. The data presented in this study are openly available in the Medline and PubMed databases and on the publisher’s website. The keywords that were used include “SARS-CoV2 infection”, “COVID-19”, “liver disease”, “Coronaviridae”, “respiratory symptoms”. All data in the text are quoted and all works used are listed in the bibliography along with DOI and reference numbers.
